# Supplementary material for: Chromothripsis during telomere crisis is independent of NHEJ, and consistent with a replicative origin
Source: Genome Res. 2019 May;29(5):737–49. doi: 10.1101/gr.240705.118 (PMC6499312; doi:10.1101/gr.240705.118)
Supplement: Supplemental Material [file supp_gr.240705.118_Supplemental_file_1.zip › contigs/annotated_contigs/DB56/contig.2.DB56_length_503_mean_cov_5.75149105368.docx]

**DB56_length_503_mean_cov_5.75149105368**

```
TGGGGTTTCACCATGTTGGCCAGGCTGGTCTTGAACTCCTGACCTCGAGTGATCCACCTGCCTCGGCCTCCCAAAGTGCTGGGATTACA
 >chr7:140969471-140969694 - E=1e-110 p=6e-03
GGTGTGAGCCACCCTGCCTGGCCGCAAAGTCACACAACTCTTATAGTAGAACTTCAGGCACCACTGCTCTCCATTCAAAAGACATAAAG

TCATCAATATCTAGGGAAATCTGTCCTGTCATGAATGTCTAGCCA|TATCAACCACCCAGTAAAAGAGACTTGATTAAATTTTTATTTG
                                               >chr14:87044482-87044762 - E=6e-157
CCAACTTAGAATGAAGAAATTTTAGAATTTTTACGTGTCTCAGAGGCATCCAGTCTGCAATATCTCCAAAGACATAAATTAATTGATTT

ATTCGACTGTTTTGTGAGCATCAAAACACAAAATACATATAGGGAATTCTAAAATAAAGATATGTTCTAGTTGTTTATATCTTATGGTA

ATTATATTTACACAAAGAAAATAACAGTCATAAAAGCTGAGGTAATGTTGAGTAAGAGT
```
